# High glucose induces Nox4 expression and podocyte apoptosis through the Smad3/ezrin/PKA pathway

**DOI:** 10.1242/bio.055012

**Published:** 2021-05-20

**Authors:** Wanxu Guo, Hang Gao, Wei Pan, Panapn Yu, Guanghua Che

**Affiliations:** 1 Department of Pediatrics, Second Hospital, Jilin University, Changchun, 130041, China; 2 The Key Laboratory of Pathobiology, Ministry of Education, Norman Bethune College of Medicine, Jilin University, Changchun 130021, China

**Keywords:** Podocyte apoptosis, High glucose, Nox4, Ezrin, PKA, Smad3

## Abstract

Podocytes are the major target in proteinuric kidney diseases such as diabetic nephropathy. The underlying molecular mechanisms by which high glucose (HG) results in podocyte damage remain unclear. This study investigated the regulatory role of Smad3, ezrin, and protein kinase A (PKA) in NADPH oxidase (Nox4) expression, reactive oxidative species (ROS) production, and apoptosis in HG-treated podocytes. A human podocyte cell line was cultured and differentiated, then treated with 30 mM HG. Apoptosis and intracellular ROS levels were assessed using TUNEL and DCF assays, respectively. Expressions of Nox4, phospho-Smad3^Ser423/425^, phospho-PKA^Thr197^, and phospho-ezrin^Thr567^ were evaluated using western blotting. ELISA was used to quantify intracellular cAMP concentration and PKA activity. Knockdown assay was used to inhibit the expressions of Smad3, Nox4, and ezrin by lentiviral shRNA. In HG-treated podocytes, the level of phospho-Smad3^Ser423/425^ and phospho-ezrin^Thr567^ was increased significantly, which was accompanied by the reduction of cAMP and phospho-PKA^Thr197^. HG-induced apoptosis was significantly prevented by the Smad3-inhibitor SIS3 or shRNA-Smad3. In podocytes expressing shRNA-ezrin or shRNA-Nox4, apoptosis was remarkably mitigated following HG treatment. HG-induced upregulation of phospho-ezrin^Thr567^ and downregulation of phospho-PKA^Thr197^ was significantly prevented by SIS3, shRNA-ezrin or shRNA-Smad3. Forskolin, a PKA activator, significantly inhibited HG-mediated upregulation of Nox4 expression, ROS generation, and apoptosis. Additionally, an increase in the ROS level was prohibited in HG-treated podocytes with the knockdown of Nox4, Smad3, or ezrin. Taken together, our findings provided evidence that Smad3-mediated ezrin activation upregulates Nox4 expression and ROS production, by suppressing PKA activity, which may at least in part contribute to HG-induced podocyte apoptosis.

## Abstract (precis)

**Summary:** The actin-membrane linker protein ezrin-related signaling plays a critical role in podocyte apoptosis through regulation of Nox4 expression and ROS production.

## INTRODUCTION

Diabetic nephropathy (DN) develops in approximately 15–20% of patients both in type 1 and type 2 diabetic mellitus, causing increased morbidity and premature mortality (Bogdanović, 2008). Although overt DN or kidney failure caused by either type of diabetes are uncommon during childhood or adolescence, diabetic kidney disease in susceptible patients almost certainly begins soon after disease onset and may accelerate during adolescence, leading to microalbuminuria or incipient DN (Bogdanović, 2008; Koulouridis, 2001). In the kidneys, glomeruli normally filter salt, water and waste products from the blood through a special filter-glomerular filtration barrier, but keep protein in the blood. When these filters are damaged, protein can leak from the blood into the urine, resulting in proteinuria, which is mainly composed of albumin (Ahn and Bomback, 2020).

The glomerular filtration barrier has three layers, including fenestrated endothelial cells, the glomerular basement membrane, and the ultimately layer-slit diaphragm. The slit diaphragm is the intercellular junction between adjacent podocyte foot processes (Ahn and Bomback, 2020). Podocyte is the major target and plays a critical role in the occurrence and development of proteinuria in diabetic kidney injury (Maestroni and Zerbini, 2008; Podgórski et al., 2019), but the underlying mechanisms by which high glucose (HG) renders podocyte damage remain elusive. Reactive oxygen species (ROS) are closely associated with the progression of kidney disease. NADPH oxidase (Nox) is a district enzymatic source of the cellular ROS, and is prominently expressed in podocytes in the kidney (Yang et al., 2018). It has been reported that Nox4-derived ROS is a key trigger of podocyte injury in several disease models such as DN (Kang et al., 2019; Wang et al., 2020). Inhibition of the Smad pathway by Smad3 knockdown significantly abrogated the increase in Nox4 expression, ROS generation, and caspase-3 activity in transforming growth factor β (TGFβ)-mediated podocyte injury (Das et al., 2014). Recent findings suggest that in colon cancer cells’ TGFβ signaling can regulate pro-apoptotic function by inhibiting the upregulation of ezrin phosphorylation in an Smad3-dependent manner (Leiphrakpam et al., 2018).

Ezrin, is a member of the ezrin/radixin/moesin (ERM) family of proteins, and plays a key role in cell-surface structure adhesion, migration, and organization (Pelaseyed and Bretscher, 2018). In the kidneys, high expression of ezrin is observed in proximal tubules and glomerular podocytes (Berryman et al., 1993). Ezrin functions not only as a scaffold for transmembrane proteins, but also as a regulator of the small GTPase such as Rho and Rac1. Loss of ezrin protects podocytes from injury-induced morphological changes (Hatano et al., 2018). An increase in intracellular cyclic AMP (cAMP), which is known to activate protein kinase A (PKA) prevented puromycin aminonucleoside (PAN)-induced apoptosis in cultured podocytes (Gu et al., 2012; Li et al., 2014). Under chronic hyperglycemia, cAMP-PKA pathway activation increased renal oxidative stress by Nox4 (Fujita et al., 2014).

Therefore, this study investigated the upstream signal that enhances Nox4 expression and ROS production by focusing on the Smad3, ezrin and the PKA pathway in order to reveal the molecular mechanisms of HG-induced podocyte apoptosis.

## RESULTS

### High glucose induces podocyte apoptosis through activation of Smad3

**Fig. 1.**
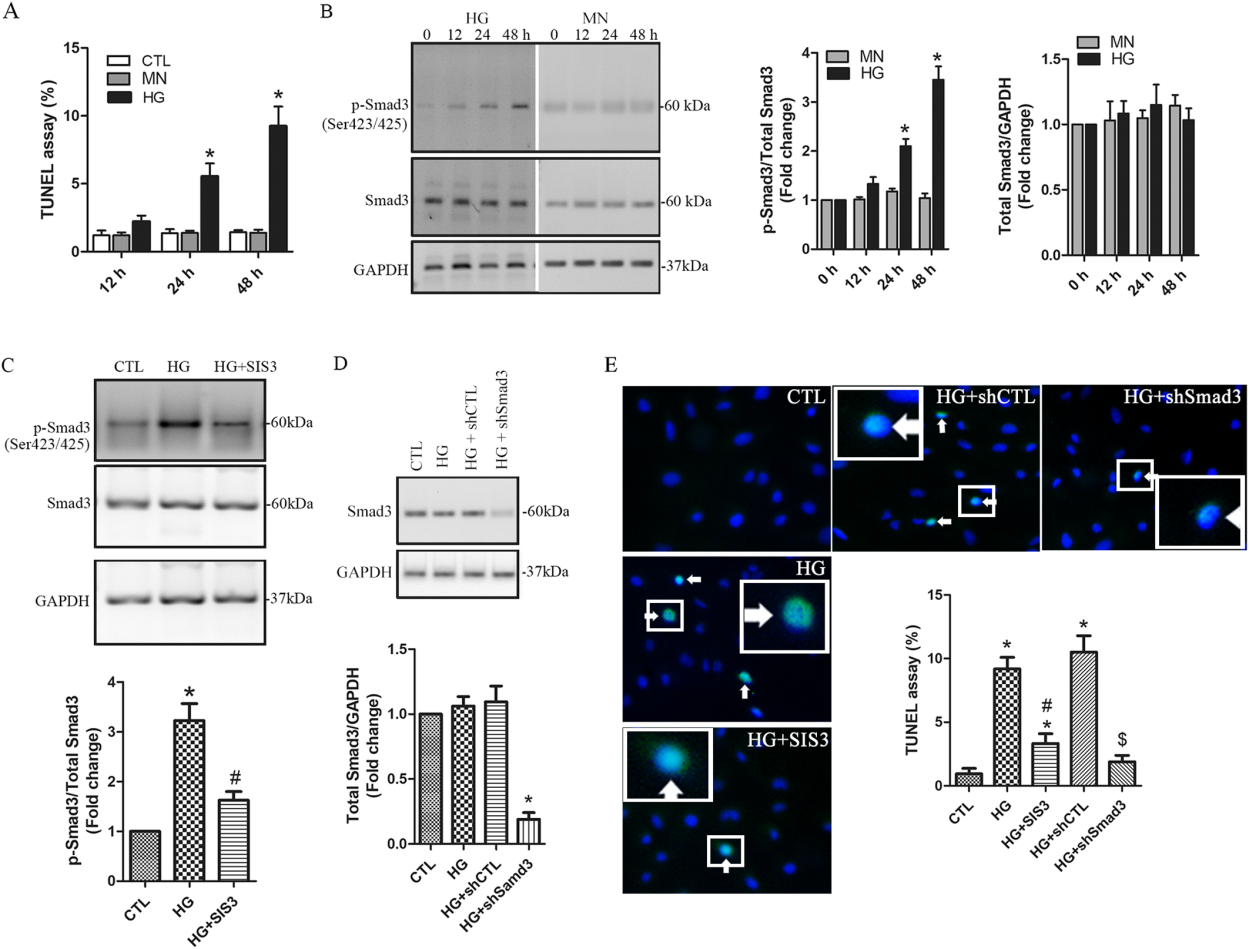
**HG induces podocyte apoptosis through activation of Smad3.** A and B. Human podocytes were treated with HG (30 mM) for 12, 24 and 48 h, respectively. Mannitol (MN) was used as the osmotic controls. Non-treated cells were used as the controls (CTL). The percentage of apoptotic cells was compared (A), and the levels of the total and the phosphorylated Smad3 were assessed using western blotting (B). *n*=3 independent experiments. Data are presented as mean±s.d. One-way ANOVA. *P<0.05 versus MN or CTL. (C) Podocytes were pretreated for 30 min with the Smad3 inhibitor SIS3 (2 µM) and then treated with 30 mM of HG for 48 h in the presence of SIS3. Non-treated cells were used as the controls (CTL). The levels of the phospho-Smad3^Ser423/425^ was assessed using western blotting. (D) Human podocytes were infected with shRNA-Smad3 (shSmad3) or the control shRNA (shCTL). After 6 h, 30 mM HG was added for 48 h. The levels of total Smad3 were evaluated using western blotting. *n=*4 independent experiments. Data are presented as mean±s.d. One-way ANOVA. *P<0.05 versus CTL, ^#^P<0.05 versus HG. (E) The effects of SIS3 and shSmad3 on apoptosis was evaluated in HG-treated podocytes. TUNEL positive cells are indicated by the arrows. A high-power view of the selected area was presented. *n*=4 independent experiments. Data are presented as mean±s.d. One-way ANOVA. *P<0.05 versus CTL, ^#^*P*<0.05 versus HG, ^$^*P*<0.05 versus HG+shCTL.

In this study, podocyte injury was assessed using TUNEL assay. Our results show that in comparison with either the non-treated or mannitol-treated cells, the percentage of TUNEL-positive cells was increased significantly at 24 and 48 h following HG treatment (Fig. 1A). Smad3 signaling plays a crucial role in induction of apoptosis in various cell types (Grynberg et al., 2017). Compared to the mannitol controls, an significant increase in phospho-Smad3^Ser423/425^ was detected at 24 and 48 h in HG-treated podocytes (Fig. 1B), indicative of activation of Smad3 signaling. To evaluate the role of the Smad3 pathway, a specific Smad3 inhibitor, SIS3, was applied to HG-treated cells. The administration of SIS3 dramatically suppressed increase in phospho-Smad3^Ser423/425^ in HG-treated podocytes (Fig. 1C). Furthermore, Smad3 expression was ablated using lentiviral shRNA-Smad3 (Fig. 1D). The data from a TUNEL assay show that the administration of either SIS3 or shRNA-Smad3 significantly decreased HG-induced apoptosis (Fig. 1E). These findings suggest that activation of Smad3 signaling involves HG-induced podocyte apoptosis.

### Smad3-mediated upregulation of the phosphorylated ezrin involves HG-induced podocyte apoptosis

**Fig. 2.**
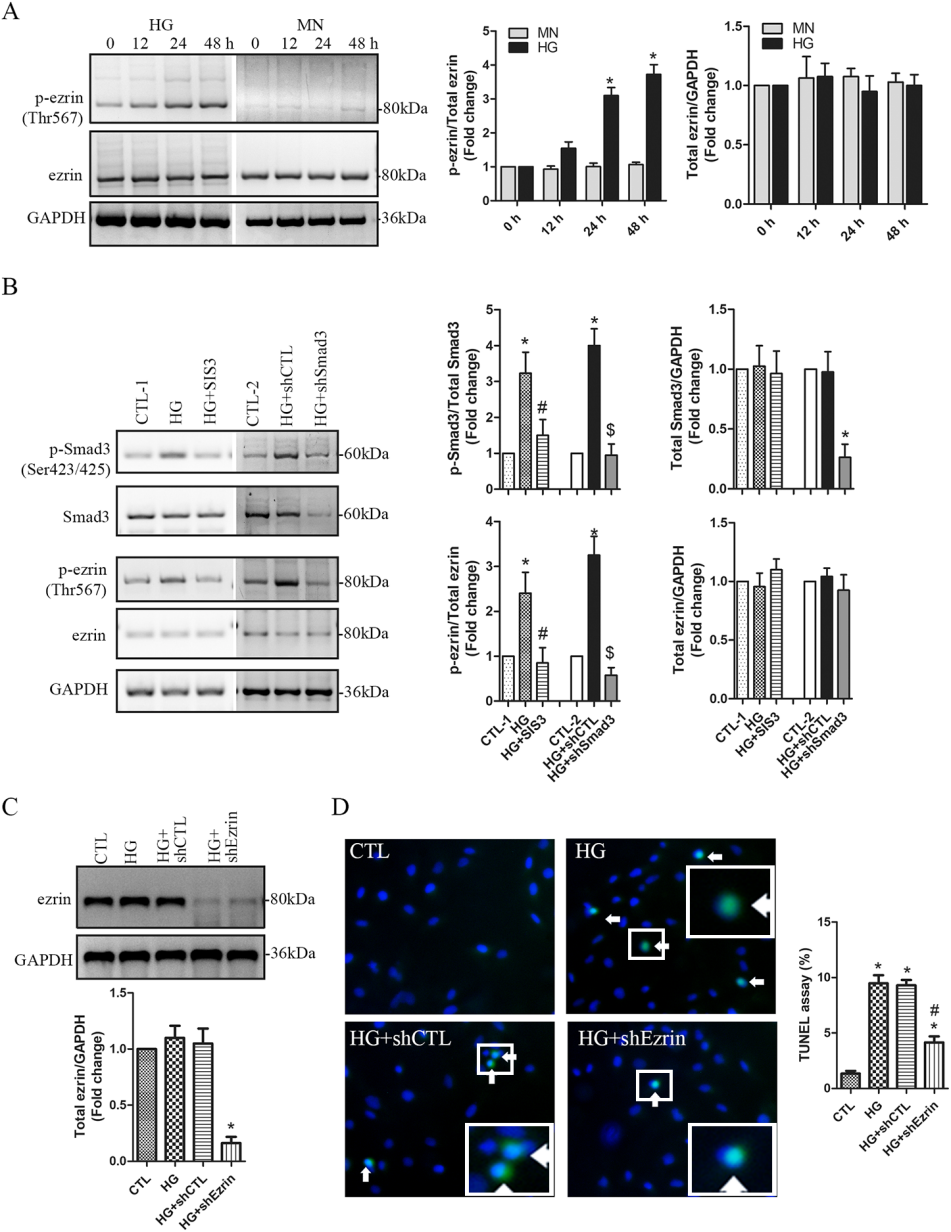
**Smad3-mediated upregulation of phosphorylated ezrin involves** **HG-induced podocyte apoptosis.** (A) Human podocytes were treated with HG (30 mM) for 0, 12, 24 and 48 h, respectively. Mannitol (MN) was used as the osmotic controls. The levels of the phosphorylated ezrin at Thr567 and total ezrin were assessed using western blotting. *n*=4 independent experiments. Data are presented as mean±s.d. One-way ANOVA. *P<0.05 versus 0 h. (B) Human podocytes were pretreated for 30 min with the Smad3 inhibitor SIS3 (2 µM) and then treated with 30 mM of HG for 48 h in the presence of SIS3. In the other set of experiment, podocytes were infected with shRNA-Smad3 (shSmad3) or the control shRNA (shCTL). After 6 h, 30 mM HG was added for 48 h. Non-treated or infected cells were used as the controls (CTL-1 or CTL-2). The levels of the phospho-Smad3^Ser423/425^ and phospho-ezrin^Thr567^ were assessed. *n*=3 independent experiments*.* Data are presented as mean±s.d. One-way ANOVA*.* *P<0.05 versus CTL-1 or CTL-2, ^#^P*<*0.05 versus HG, ^$^*P*<0.05 versus HG+shCTL*.* (C,D) Human podocytes stably expressing shRNA-Ezrin (shEzrin) or the control shRNA (shCTL) were treated for 48 h with 30 mM of HG. Non-treated cells were used as the controls (CTL). The protein levels of total ezrin was assessed (C). The effect of ezrin knockdown on apoptosis was evaluated using the TUNEL assay (D). TUNEL positive cells are indicated by the arrows. A high-power view of the selected area was presented. *n*=4 independent experiments. Data are presented as mean±s.d. One-way ANOVA. *P<0.05 versus 0 h or CTL, ^#^*P*<0.05 versus HG.

Ezrin, also known as cytovillin or villin-2, is a cytoplasmic peripheral protein containing a FERM domain on its N-terminus and an ERM domain on its C-terminus (Pelaseyed and Bretscher, 2018). Phosphorylation of ezrin on threonine 567 (Thr567) in the C-terminal domain stabilizes the protein's conformation in its active mode (Pelaseyed and Bretscher, 2018). In HG-treated podocytes, phosphor-ezrin^Thr567^ was upregulated significantly at 24 and 48 h compared to the mannitol controls (Fig. 2A). To reveal if HG activated ezrin through Smad3 signaling, we inhibited Smad3 signaling using SIS3 or shRNA-Smad3. Our results show that both inhibition and knockdown of Smad3 remarkably abrogated upregulation of phospho-ezrin^Thr567^ in HG-treated podocytes (Fig. 2B). Ezrin plays a key role in cellular survival, proliferation, and apoptosis (Leiphrakpam et al., 2018). In this study, ezrin expression was significantly knocked down using lentiviral shRNA-ezrin (Fig. 2C). In comparison with the control shRNA, shRNA-ezrin significantly alleviated HG-induced apoptosis (Fig. 2D). These findings indicate that Smad3-mediated activation of ezrin plays a pivotal role in HG-induced podocyte apoptosis.

### Activated ezrin downregulates PKA activity in HG-treated podocytes

**Fig. 3.**
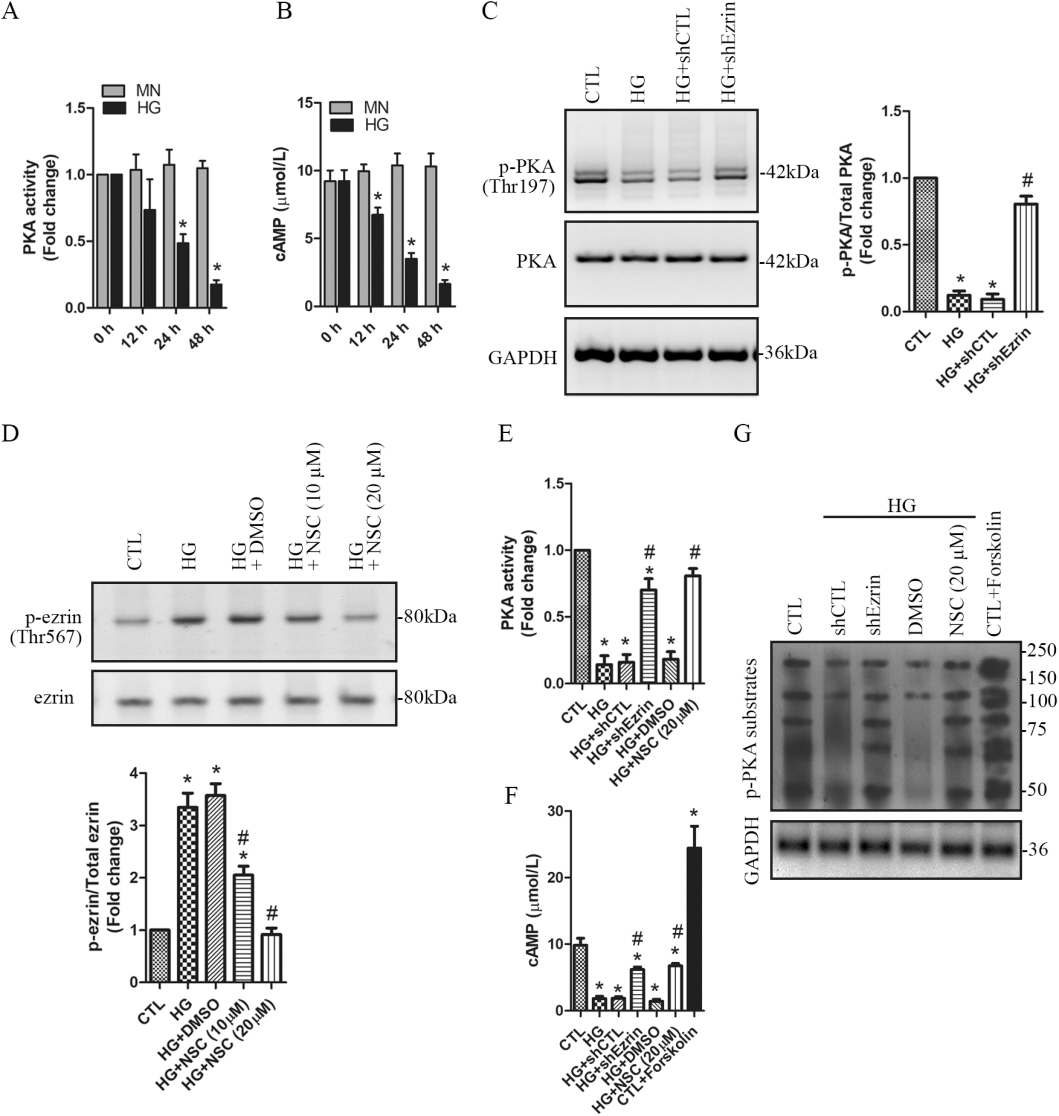
**HG** **inhibits PKA activation through downregulation of cAMP by the phosphorylated ezrin in podocytes.** (A,B) Human podocytes were treated with HG (30 mM) for 0, 12, 24 and 48 h, respectively. Mannitol (MN) was used as the osmotic controls. PKA activity (A) and intracellular cAMP levels (B) were measured and compared. *n*=4 independent experiments. Data are presented as mean±s.d. One-way ANOVA. *P<0.05 versus 0 h. (C) Human podocytes stably expressing shRNA-Ezrin (shEzrin) or the control shRNA (shCTL) were treated for 48 h with 30 mM of HG. The protein levels of the phospho-PKA^Thr197^ were assessed using western blotting. (D) Human podocytes were pretreated for 30 min with the ezrin inhibitor NSC668394 (NSC: 10 µM or 20 µM) or DMSO, and then treated with 30 mM of HG for 48 h in the presence of NSC668394 or DMSO. Non-treated cells were used as the controls (CTL). The levels of the phospho-ezrin^Thr567^ were assessed using western blotting. *n*=3 independent experiments. Data are presented as mean±s.d. one-way ANOVA. *P<0.05 versus CTL, ^#^*P*<0.05 versus HG or HG+DMSO. (E) The effects of ezrin knockdown and NSC668394 on PKA activity were evaluated. (F) The effects of ezrin knockdown and NSC668394 on intracellular cAMP levels were evaluated. Podocytes treated with forskolin (20 µM) alone were used as the positive control for cAMP generation. *n*=3 independent experiments. Data are presented as mean±s.d. one-way ANOVA. *P<0.05 versus CTL, ^#^*P*<0.05 versus HG, HG+shCTL, or HG+DMSO. (G) The phosphor-PKA substrates were assessed using western blotting in HG-treated podocytes with ezrin knockdown (shEzrin) or ezrin inhibition (NSC668394). Podocytes treated with forskolin (20 µM) were used as the positive control for cAMP generation and PKA activation.

PKA, a cAMP-dependent protein kinase, has several functions in the cell, including regulation of glycogen, sugar, and lipid metabolism (Braun et al., 2018). PKA has two catalytic and two regulatory sub-units. Bind of cAMP to the regulatory sub-units causes them to break apart from the catalytic sub-units, leading to activation of PKA (Cheng et al., 2008). In this study, PKA activity and intracellular cAMP concentration were measured and compared in HG-treated podocytes. Our data show that HG treatment time-dependently decreased PKA activity and cAMP level (Fig. 3A,B). It has been reported that differential status of ezrin phosphorylation regulates PKA activity in the process of TGFβ and insulin-like growth factor 1 receptor (IGF1R)-induced signaling cascades (Leiphrakpam et al., 2018). In our study, ezrin knockdown significantly elevated PKA activity in HG-treated podocytes (Fig. 3C). Notably, there is the possibility that specific interactors with the ‘closed’ or inactive conformation of ezrin are also altered when an ezrin knockdown is performed (Viswanatha et al., 2013). To further demonstrate the role of phospho-ezrin in suppressing PKA activity, we applied NSC668394, a specific ezrin phosphorylation inhibitor to abrogate ezrin phosphorylation at Thr567 (Fig. 3D). ELISA assay shows that both ezrin knockdown and NSC668394 significantly prevented HG-induced reduction of PKA activity (Fig. 3C) and the intracellular cAMP concentration (Fig. 3F). Inconsistent reduction of the phosphorylated PKA substrates was detected in HG-treated podocytes, which was significantly prevented by ezrin knockdown or NSC668394 (Fig. 3G). These data imply that HG treatment decreased PKA activity potentially through phospho-ezrin^Thr567^ in podocytes.

### HG-induced podocyte apoptosis is suppressed by upregulation of PKA activity

**Fig. 4.**
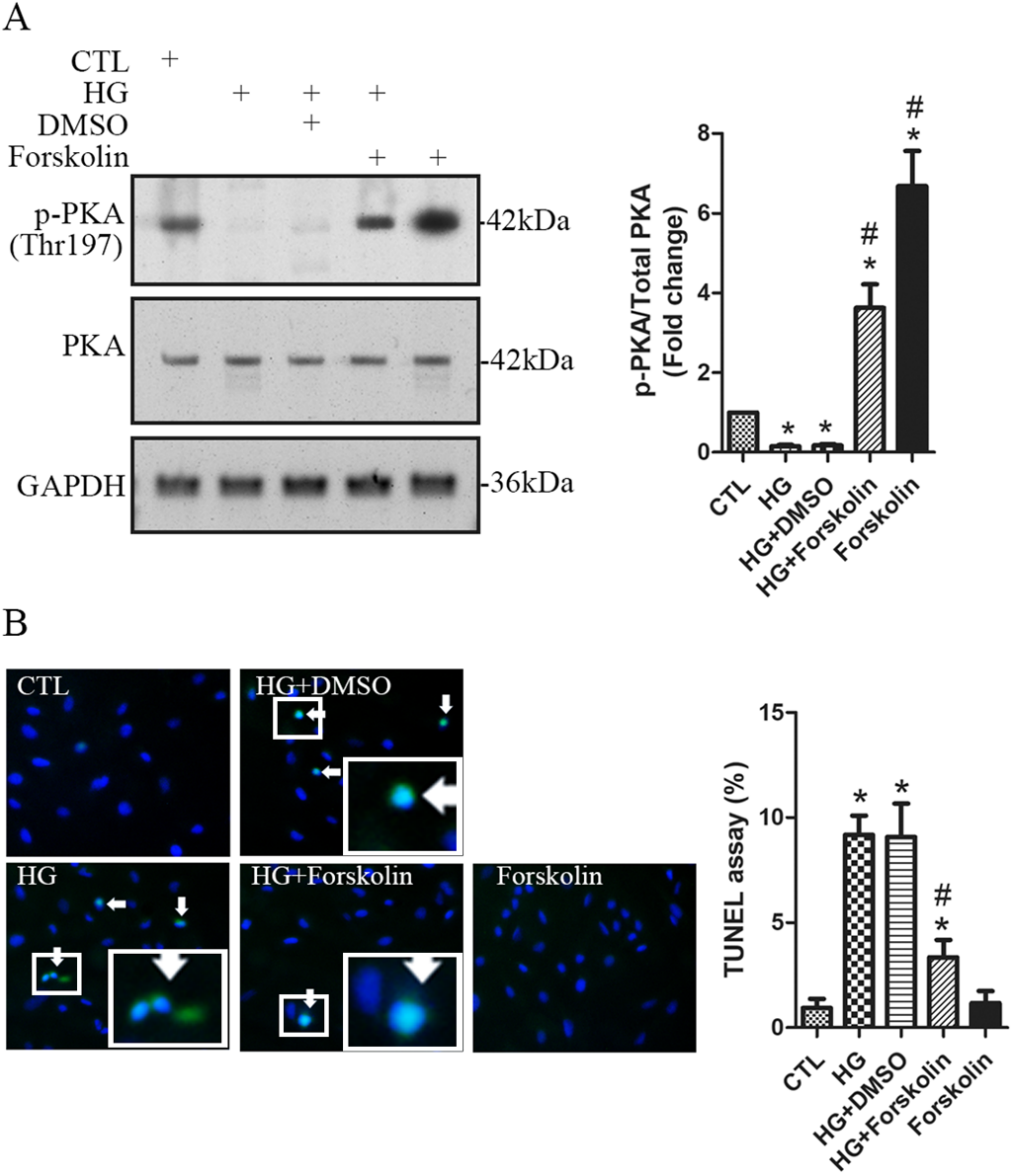
**HG-induced podocyte apoptosis is inhibited by upregulating PKA activation levels.** Podocytes were pretreated for 30 min with the PKA activator forskolin (20 µM) or DMSO, and then treated with 30 mM of HG for 48 h in the presence of forskolin or DMSO. Non-treated cells were used as the controls (CTL). (A) The levels of the phospho-PKA^Thr197^ were assessed using western blotting. (B) Effect of forskolin on apoptosis was evaluated using the TUNEL assay. TUNEL positive cells are indicated by the arrows. A high-power view of the selected area was presented. *n=*4 independent experiments. Data are presented as mean±s.d. one-way ANOVA. *P<0.05 versus CTL, ^#^*P*<0.05 versus HG or DMSO.

Forskolin is a specific PKA activator, which activates PKA by increasing intracellular cAMP generation (Chung et al., 2015). In non-treated podocytes, forskolin significantly increased the intracellular cAMP concentration and the phosphorylated PKA substrates (Fig. 3F,G). To investigate the role of PKA in HG-induced podocyte apoptosis, we applied forskolin to HG-treated cells. In non-treated podocytes, forskolin significantly increased phospho-PKA^Thr197^ (Fig. 4A), suggesting that forskolin worked well in upregulating PKA activity. In HG-treated podocytes, the reduction of phospho-PKA^Thr197^ was detected, which was significantly prevented by forskolin (Fig. 4A). We then evaluated the effects of forskolin on apoptosis. Forskolin alone did not show significant influence on apoptosis (Fig. 4B). However, HG-induced apoptosis was significantly decreased by forskolin (Fig. 4B). These data suggest that HG-induced podocyte apoptosis was alleviated by upregulation of PKA activity.

### HG induces apoptosis by upregulating Nox4 expression via Smad3/ezrin-mediated suppression of PKA activity in podocytes

**Fig. 5.**
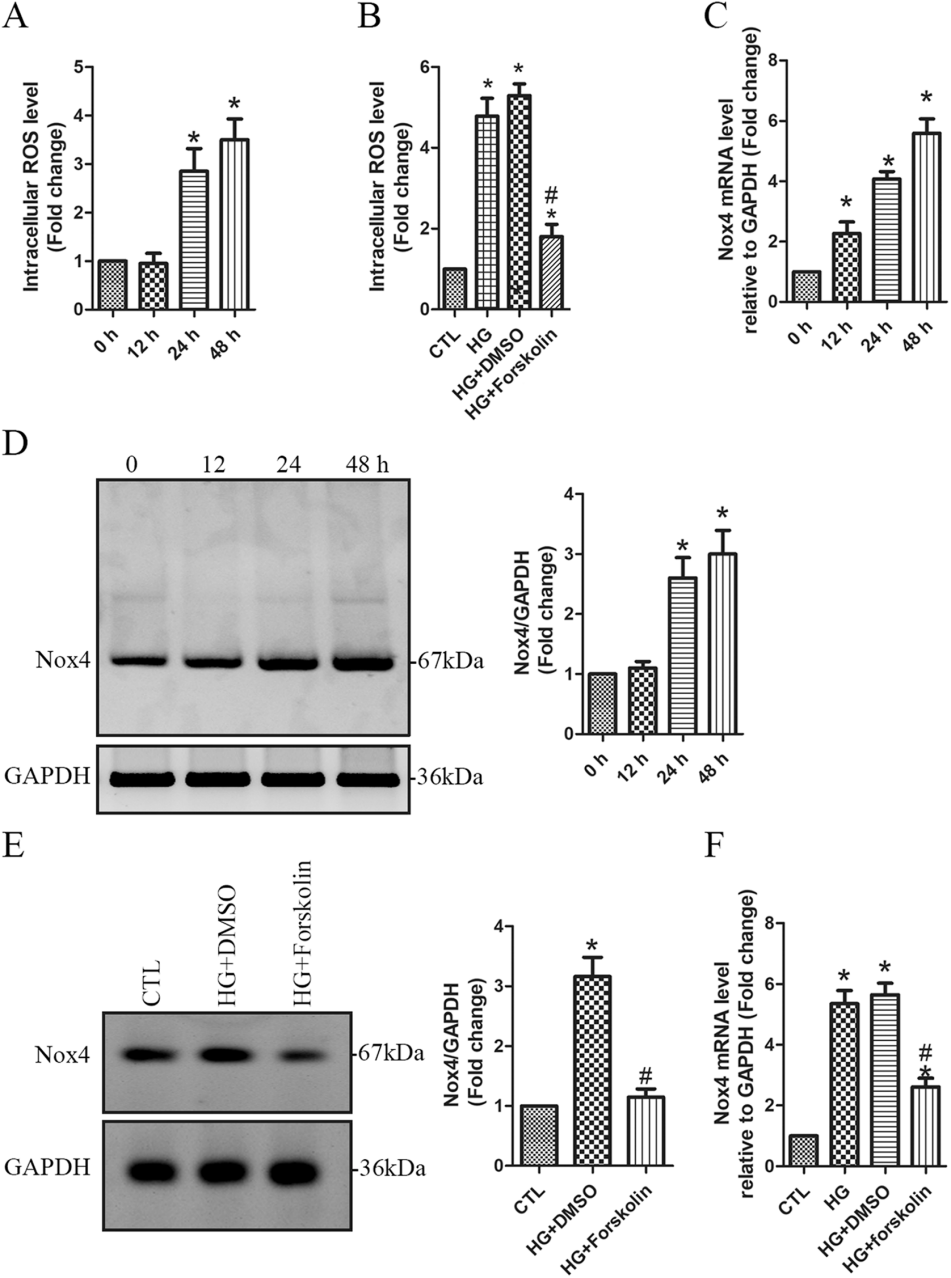
**HG-induced upregulation of Nox4 expression is inhibited by the PKA activator forskolin.** (A) Human podocytes were treated with HG (30 mM) for 0, 12, 24 and 48 h, respectively. The intracellular ROS level was measured using DCF assay. *n=*4 independent experiments. Data are presented as mean±s.d. one-way ANOVA. *P<0.05 versus 0 h. (B) Podocytes were pretreated for 30 min with the PKA activator forskolin (20 µM) or DMSO, and then treated with 30 mM of HG for 48 h in the presence of forskolin or DMSO. Non-treated cells were used as the controls (CTL). The intracellular ROS level was measured. *n*=4 independent experiments. Data are presented as mean±s.d. one-way ANOVA. *P<0.05 versus CTL, ^#^*P*<0.05 versus HG or HG+DMSO. (C,D) Podocytes were treated with HG (30 mM) for 0, 12, 24 and 48 h, respectively. Nox4 mRNA (C) and protein (D) level was assessed using qPCR and western blotting, respectively. *n*=4 independent experiments. Data are presented as mean±s.d. one-way ANOVA. *P<0.05 versus 0 h. (E,F) Podocytes were pretreated for 30 min with the PKA activator forskolin (20 µM) or DMSO, and then treated with 30 mM of HG for 48 h in the presence of forskolin or DMSO. Non-treated cells were used as the controls (CTL). Nox4 protein (E) and mRNA (F) level was assessed using western blotting and qPCR, respectively. *n*=4 independent experiments. Data are presented as mean±s.d. one-way ANOVA. *P<0.05 versus CTL, ^#^*P*<0.05 versus HG or HG+DMSO.

It has been demonstrated that HG causes podocyte apoptosis predominantly through Nox4-mediated increase of ROS production (Kang et al., 2019; Wang et al., 2020). As such, we measured the intracellular ROS level, which showed a significant increase at 24 and 48 h following HG treatment (Fig. 5A). In HEK293 cells, it has been reported that Nox-dependent ROS production was reduced by elevating cAMP generation and activating PKA (Kim et al., 2007). Interestingly, HG-induced increase of ROS was significantly prevented by the PKA activator forskolin (Fig. 5B), suggesting that PKA may have a regulatory role in ROS production. We thereafter assessed Nox4 expression both at the mRNA and the protein levels. qPCR shows a rapid increase of Nox4 mRNA level from 12 h persisting to 48 h following HG treatment (Fig. 5C). Western blotting shows that Nox4 protein levels were significantly increased at 24 and 48 h in HG-treated podocytes (Fig. 5D). Furthermore, our data show that forskolin significantly suppressed HG-induced upregulation of Nox4 expression both at the protein (Fig. 5E) and the mRNA levels (Fig. 5F). These findings suggest that the cAMP-PKA pathway appears to play an important inhibitory role in regulation of Nox4 expression. In HG conditions, PKA activity is decreased, and thus Nox4 expression is upregulated due to loss of inhibitory regulation of PKA.

**Fig. 6.**
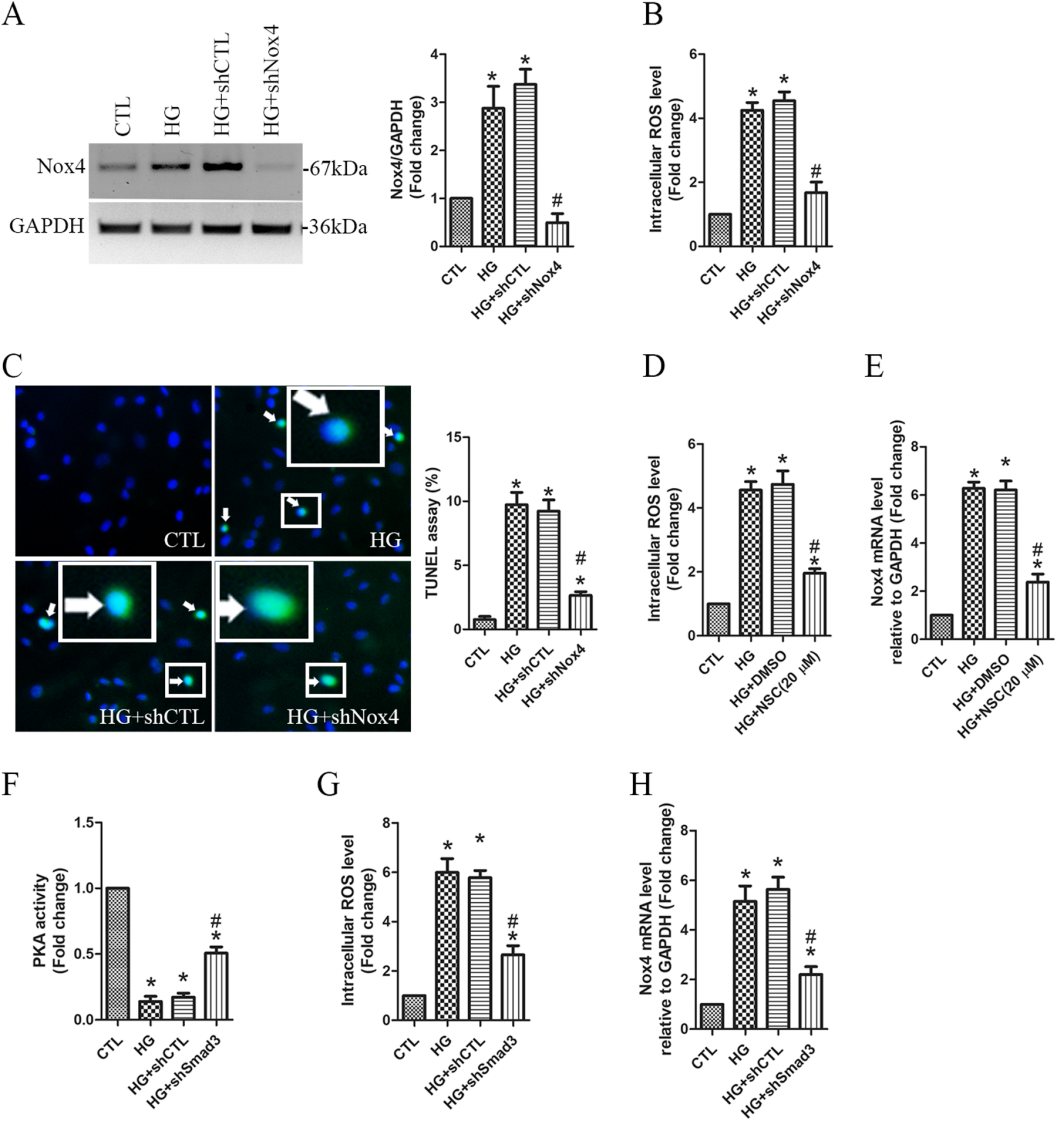
**HG** **induces podocyte apoptosis through upregulation of Nox4 by regulation of the Smad3/ezrin/PKA signaling.** Human podocytes stably expressing shRNA-Nox4 (shNox4) or the control shRNA (shCTL) were treated for 48 h with 30 mM HG. (A) Nox4 protein level was evaluated using western blotting. (B) Effects of Nox4 knockdown on the ROS level were assessed using DCF assay. (C) Effects of Nox4 knockdown on apoptosis were assessed using TUNEL assay. TUNEL positive cells are indicated by the arrows. A high-power view of the selected area was presented. *n*=4 independent experiments. Data are presented as mean±s.d. one-way ANOVA. *P<0.05 versus CTL, ^#^*P*<0.05 versus HG or HG+shCTL. (D,E) Human podocytes were pretreated for 30 min with the ezrin inhibitor NSC668394 (NSC: 20 µM) or DMSO, and then treated with 30 mM of HG for 48 h in the presence of NSC668394 or DMSO. Non-treated cells were used as the controls (CTL). Effects of NSC668394 on intracellular ROS level (D) and Nox4 mRNA expression (E) were evaluated using DCF and qPCR, respectively. *n*=3 independent experiments. Data are presented as mean±s.d. one-way ANOVA. *P<0.05 versus CTL, ^#^*P*<0.05 versus HG or HG+DMSO. (F-H) Human podocytes were infected with shRNA-Smad3 (shSmad3) or the control shRNA (shCTL). After 6 h, 30 mM HG was added for 48 h. Effects of Smad3 knockdown on PKA activation level was evaluated using PKA assay (F). Effects of Smad3 knockdown on intracellular ROS level (G) and Nox4 mRNA expression (H) were evaluated using DCF and qPCR, respectively. *n*=3 independent experiments. Data are presented as mean±s.d. one-way ANOVA. *P<0.05 versus CTL, ^#^*P*<0.05 versus HG or HG+shCTL.

We then assessed the role of Nox4 in increase in the ROS generation in HG-treated podocytes. We created stable Nox4-knockdown podocyte cell line with lentiviral shRNA-Nox4. Nox4 protein levels were dramatically abrogated in HG-treated podocytes stably expressing shRNA-Nox4, but not in those expressing the control shRNA (Fig. 6A). Moreover, HG-induced ROS overproduction was significantly prevented by Nox4 knockdown (Fig. 6B). The percentage of apoptotic cells was significantly decreased by Nox4 knockdown in HG-treated podocytes (Fig. 6C). These data suggest that Nox4-dependent increase in ROS plays a key role in HG-induced podocyte apoptosis.

**Fig. 7.**
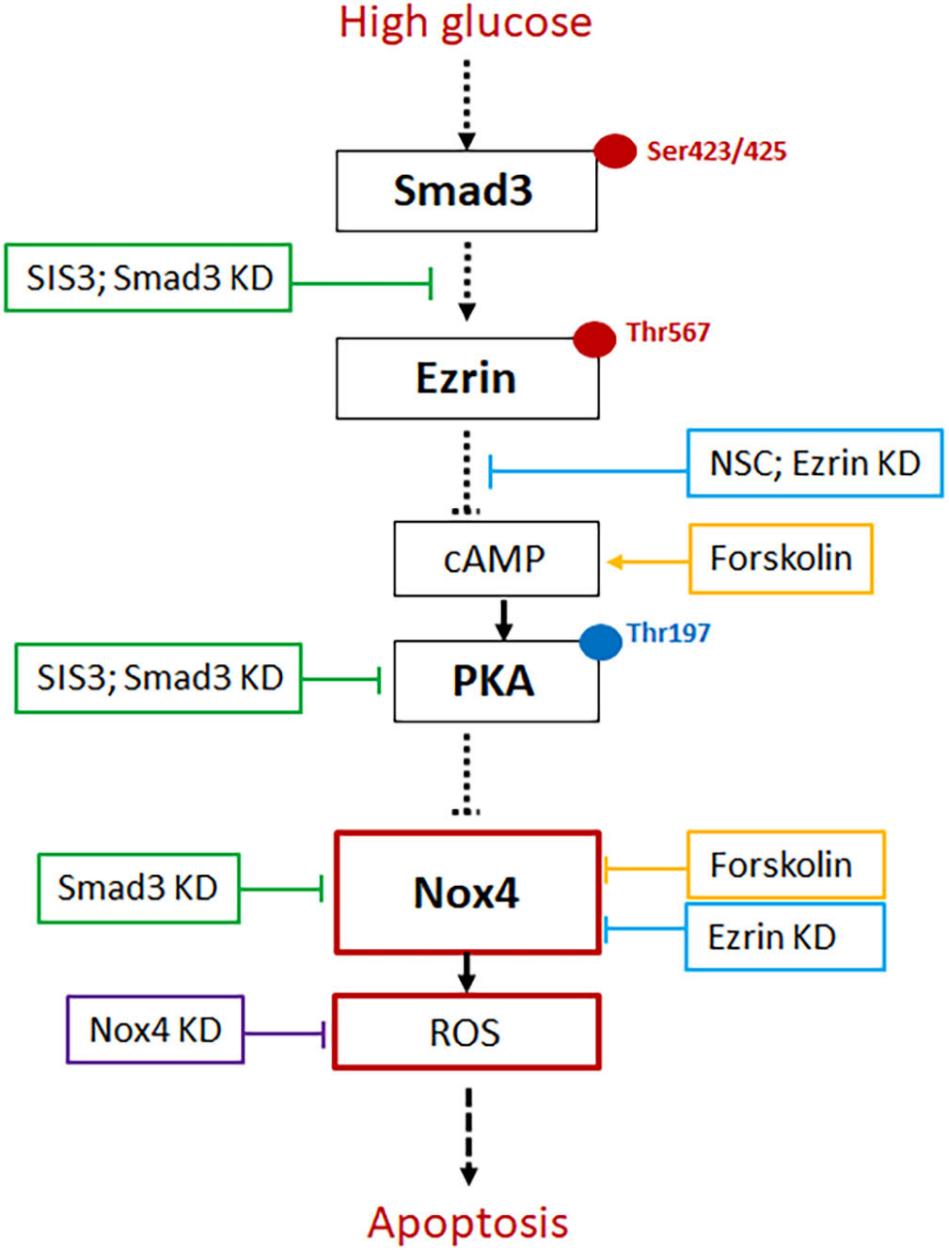
**Proposed mechanism of high glucose-induced podocyte apoptosis by the Smad3/ezrin/PKA/Nox4 pathways.** High glucose treatment enhances ezrin phosphorylation on Thr567 by activating Smad3 on Ser423/425, which contributes to Nox4 upregulation, intracellular ROS overproduction, and podocyte apoptosis, by inhibiting cAMP-mediated PKA phosphorylation on Thr197. SIS3: Smad3 inhibitor, KD: knockdown with lentiviral shRNA, NSC: NSC668394, ezrin phosphorylation inhibitor, Forskolin: PKA activator by increasing cAMP generation, ROS: reactive oxidative species, Nox4: NADPH oxidase 4.

Additionally, our data show that NSC668394, an ezrin phosphorylation inhibitor, significantly prevented HG-induced increase in ROS generation (Fig. 6D) and Nox4 mRNA expression (Fig. 6E). Furthermore, our data indicated that in HG-treated podocytes, Smad3 knockdown dramatically increased PKA activity (Fig. 6F), but decreased the ROS level (Fig. 6G) and Nox4 mRNA expression (Fig. 6H). These findings suggest that Smad3/ezrin-mediated suppression of PKA activity at least in part involves HG-induced upregulation of Nox4 and ROS, being responsible for induction of apoptosis (Fig. 7).

## DISCUSSION

Nox4, the predominant Nox isoform expressed in the kidneys, is a major source of superoxide. Expression of Nox4 is increased in podocytes cultured in HG conditions and in the kidney tissues from both DN patients and rodent models (Meng et al., 2018). It has been found that the intracellular ROS level was increased significantly in podocytes in DN (Eid, 2009; Li et al., 2019). In the present study, we have demonstrated that Nox4 expression and the intracellular ROS level were time-dependently increased following HG treatment in the cultured human podocytes. Moreover, Nox4 knockdown significantly suppressed HG-mediated increase in ROS generation and apoptosis, indicating that HG causes podocyte apoptosis at least partially in the Nox4-dependent ROS pathway.

Previous studies have shown that activated PKA signaling prevented podocyte apoptosis by promoting mitochondrial fusion (Kim et al., 2007). Glucagon-like peptide-1 (GLP-1) is a gut incretin hormone, and has an antioxidative protective effect on various tissues. It has been reported that GLP-1 has a crucial role in protection against increased ROS under chronic hyperglycemia, by inhibition of Nox4 and by activation of the cAMP-PKA pathway (Fujita et al., 2014). Intracellular cAMP activates PKA by binding to its regulatory sub-units and causing them to dissociate from the catalytic sub-units (Cheng et al., 2008). In our study, a significant reduction in phosphorylated PKA on Thr197 was detected in HG-treated podocytes, which was accompanied by a decrease in intracellular cAMP concentration. These findings suggest that HG resulted in suppression of PKA activity in podocytes. To prevent reduction of PKA activity, we applied forskolin, a specific PKA activator which can activates PKA by increasing intracellular cAMP generation (Chung et al., 2015). We found that HG-induced reduction in PKA activity and cAMP concentration was remarkably prevented by forskolin. Moreover, the administration of forskolin significantly decreased HG-induced apoptosis. In PAN-treated podocytes, the cAMP signaling prevented podocyte apoptosis via activation of PKA and mitochondrial fusion (Gu et al., 2012; Li et al., 2014). Furthermore, our results showed that HG-induced increase of Nox4 expression and ROS was significantly suppressed by forskolin, suggesting that HG-induced Nox4 expression was possibly caused by inhibition in PKA activity in podocytes. Nevertheless, other factors downstream of PKA could also be involved in apoptosis regulation in parallel to the Smad3 signaling. As such, if and how Nox4 expression is regulated by PKA signaling in HG-induced podocyte injury needs be further investigated. Our recent findings demonstrated that Nox4-dependent ROS production is responsible for apoptosis through a small GTPase Arf6-mediated Erk1/2 activation in angiotensin II (Ang II)-treated podocytes (Che et al., 2020). The mammalian target of the rapamycin (mTOR) signaling cascade controls cellular metabolism, survival and growth. It has been reported that mTORC2 plays a critical role in Nox4-derived ROS generation and podocyte apoptosis, which contributes to urinary albumin excretion in type 1 diabetes (Eid et al., 2016). In addition, podocyte-specific knockout of β-catenin protected mice against podocyte injury and albuminuria after treatment with advanced glycation end products. The receptor of advanced glycation end products (RAGE)-mediated Nox2 induction and ROS generation, resulted in upregulation of Wnt/β-catenin via nuclear factor-κB activation (Zhou et al., 2019). The endoplasmic reticulum (ER) is a highly dynamic organelle responsible for multiple cellular functions. ER stress is common under various pathogenic microenvironments and contributes to progression of various podocyte diseases. It has been found that PAN-induced ER stress increased Nox4-related oxidative stress and apoptosis (Min et al., 2018). Therefore, the regulatory mechanisms of Nox expression and the crosstalk among different signal pathways need to be further investigated, in order to address its distinct role in podocyte injury in diabetes.

Ezrin is a member of the ezrin/radixin/moesin (ERM) family of proteins, which function as general cross-linkers between plasma membrane proteins and the actin cytoskeleton and are involved in the functional expression of membrane proteins at the cell surface (Gallop, 2020). In ezrin knockout mice, loss of ezrin protects podocytes from lipopolysaccharide (LPS)-induced morphological changes by suppressing Rac1 activation (Hatano et al., 2018). In colon cancer cells, TGFβ and IGF1R signaling activates PKA through regulation of ezrin phosphorylation (Leiphrakpam et al., 2018). Some studies have shown that ezrin can anchor and form a complex with PKA, and then phosphorylates serine 369 and 373 on connexin 43, to enhance gap-junction assembly, communication, and cell fusion (Dukic et al., 2018). These studies suggest that ezrin can regulate PKA activation status in multiple cell types. In our study, the levels of the phosphorylated ezrin^Thr567^ were increased significantly, but total ezrin abundance did not change following HG treatment. However, abundance of ezrin was reduced at 4 weeks after induction of diabetes by streptozotocin in rats. Of note, albuminuria and high blood glucose were detected, but streptozotocin-injected rats did not yet show ultrastructural changes in glomeruli (Wasik et al., 2014). These findings might reflect the change of ezrin behavior at an early stage of diabetic disease. In addition, downregulation of ezrin activation at Thr567 was detected in this diabetic model. Nevertheless, it is unclear whether decrease of ezrin activation was caused by its abundance reduction. The dynamic alterations of ezrin expression and its activation status as well as the role of ezrin in the pathogenesis of diabetic disease should be further investigated and clarified. Our data showed that ezrin knockdown or inhibition dramatically increased the intracellular cAMP concentration and enhanced PKA activity, but suppressed HG-induced apoptosis. This suggests that HG treatment mediates podocyte apoptosis, potentially through suppression of PKA by ezrin activation. Some studies have shown beneficial effects of all-trans-retinoic acid (ATRA) on HIV-induced podocyte injury, which are mediated through the activation of the PKA pathway and the reduction of MAPK1,2 phosphorylation (Chuang and He, 2009), indicative of a crosstalk between the PKA and MAPK pathways. The role of PKA-related signaling in the process of podocyte injury needs to be explored. It has been found that in response to insulin stimulation, ezrin and phosphorylated ezrin translocated to the cell periphery in cultured mouse podocytes, and knockdown of ezrin increased glucose uptake by increasing translocation of glucose transporter GLUT1 to the plasma membrane (Wasik et al., 2014). Cofilin-1 is essential for the turnover and reorganization of actin filaments (Berger and Moeller, 2011). Phosphorylated, inactive cofilin-1 was upregulated in glomeruli isolated from streptozotocin-induced diabetic rats (Wasik et al., 2014). Moreover, knockdown of ezrin resulted in actin remodeling by increasing cortical actin formation under basal conditions, but reduced insulin-stimulated actin reorganization by affecting cofilin-1 location (Wasik et al., 2014). These findings suggest that other pathways such as glucose uptake and cofilin-related actin remodeling could also involve ezrin-induced signaling in HG-mediated podocyte apoptosis, not only Smad and/or PKA dependent. Additionally, the actin cytoskeleton has been implicated in regulating apoptosis signaling (Desouza et al., 2012). Ezrin, the actin-membrane linker protein, acts as a mediator of CD95-mediated apoptosis in human T lymphocytes (Parlato et al., 2000). In lymphoma cells, translocation of oxidized cofilin to the mitochondria can induce the release of cytochrome c due to mitochondrial membrane permeabilization, resulting in apoptosis (Klamt et al., 2009).

TGFβ and Smad3 signaling plays a crucial role in the pathogenesis of diabetic kidney injury (Lan and Chung, 2011). Podocyte injury was alleviated in obese Smad3-deficient mice (Sun et al., 2015). In cultured mouse podocytes, Smad3 signaling affects HG-induced podocyte injury via regulation of the cytoskeletal protein transgelin (Jiang et al., 2020). We found that Smad3 was remarkably activated at Ser423/425 in HG-treated podocytes, and inhibition of Smad3 by its specific inhibitor SIS3 or shRNA-Smad3 significantly decreased HG-induced apoptosis. Moreover, the application of SIS3 or Smad3 knockdown significantly suppressed the phosphorylation of ezrin, but upregulated PKA activity. In lung adenocarcinoma, tumor-associated macrophages promote ezrin-phosphorylation-mediated epithelial-mesenchymal transition through fucosyltransferase IV-induced fucosylation, in which upregulation of phospho-Smad3 plays a pivotal role (LaGier et al., 2007). These data suggest that activation of the Smad3/ezrin signaling plays an important role in suppression of PKA activity in HG-treated podocytes.

Taken together, we provide evidence that following HG treatment, Smad3 is activated, leading to increase of phosphorylated ezrin, which upregulates Nox4 expression, ROS production, and apoptosis by suppressing PKA activity. Of note, it would be possible that upon activation, Smad3 on its own is capable of translocating to the nucleus and inducing Nox4 expression by binding to the promotor, without the direct involvement of PKA and Ezrin. Due to the complexity of signaling cascades, it remains unclear if the correlation between Smad3-ezrin-PKA-Nox4 is in fact causal and linear. In addition, a wide variety of pathways such as ER stress, mitochondrial dysfunction, mTOR, Erk1/2, and MAPK involve podocyte injury. The challenge for future studies is to differentiate and identify key signaling pathways that are responsible for podocyte injury and the pathogenesis of proteinuria in diabetic kidney disease.

## MATERIALS AND METHODS

### Antibodies

The primary antibodies used in this study include rabbit anti-phospho-Smad3^Ser423/425^ (#9520, 1:500; Cell Signaling Technology, Danvers, MA, USA), rabbit anti-Smad3 (#9513, 1:1000; Cell Signaling Technology), rabbit anti-phospho-ezrin^Thr567^ (PA5-37763, 1:500; Invitrogen, Carlsbad, CA, USA), mouse anti-ezrin (#35-7300, 1:1000; Invitrogen), rabbit anti-phospho-PKA α/β CAT (pThr197) (SAB4301240, 1:500; Sigma-Aldrich, Burlington, MA, USA), rabbit anti-PKA (06-903, 1:800; Sigma-Aldrich), rabbit anti-phospho-PKA substrates antibody (#9624, 1:1000; Cell Signaling Technology), rabbit anti-NADPH oxidase 4 (ab133303, 1:750; Abcam, Cambridge, MA, USA), and mouse anti-GAPDH (G8795, 1:10,000; Sigma-Aldrich) antibodies.

### Podocyte culture and treatment

As described previously (Saleem et al., 2002), conditionally immortalized human podocytes (a gift from Professor Moin Saleem, University of Bristol, Bristol, UK) were propagated at 33°C in RPMI 1640 media (Invitrogen, Carlsbad, CA, USA) containing 10% fetal calf serum (FCS, Invitrogen), 1x Insulin-Transferrin-Selenium media supplement (Gibco, Gaithersburg, MD, USA) and 1% Pen/Strep (Invitrogen). Contamination is routinely tested and excluded in the lab. When cells grew to 60–80% confluence, they were split and cultured at 37°C in 2% FCS for 14 days for differentiation. All experiments were performed on human podocyte cell line in passages 6–10, and differentiated cells were used in this study.

Differentiated podocytes were cultured for the indicated time periods (0, 12, 24 or 48 h) in the presence of 30 mM glucose (cat. no. G8270, Sigma-Aldrich) or mannitol as an osmotic control (cat. no. PHR1007; Sigma–Aldrich). In experiments using the Smad3 inhibitor SIS3 (2 µM, cat. no. 15945, Cayman Chemical, Ann Arbor, MI, USA), the ezrin phosphorylation inhibitor NSC668394 (10 or 20 µM, cat. no. 341216, Sigma-Aldrich), the PKA activator forskolin (20 µM; cat. no. F6886, Sigma-Aldrich), cells were pretreated for 30 min with the indicated inhibitor or activator followed by HG treatment in the presence of inhibitor or activator.

### Knockdown of Smad3, ezrin, and Nox4

To knockdown expression of Nox4, ezrin and Smad3, human podocytes were infected respectively with the lentiviral shRNA particles against human Nox4 (cat. no. TRCN0000046091, Sigma-Aldrich), ezrin (cat. no. TRCN0000380178, Sigma-Aldrich), and Smad3 (cat. no. sc-38376-v, Santa Cruz Biotechnology, Shanghai, China) according to the manufacturer's protocols. The sequence of the control-shRNA (cat. no. SHC016V, Sigma-Aldrich) does not match any human genes. Stable knockdown podocyte cell line was created by addition of puromycin (final concentration 2.5 μg/ml; Sigma-Aldrich). After two weeks, puromycin-resistant cells were obtained, and knockdown efficiency was evaluated using western blotting.

### TUNEL assay

As described previously (Che et al., 2020), apoptotic cell death was assessed with the Fluorescein *In-Situ* Cell Death Detection Kit (Sigma-Aldrich) according to the manufacturer's procedure. Nuclei were stained with the 4′,6-diamidino-2-phenylindole (DAPI). The TUNEL-positive nuclei appeared as green under an immunofluorescence microscope (Zeiss, Beijing, China). The percentage of TUNEL-positive cells was calculated and compared.

### RT-qPCR

Total RNA was extracted with Trizol reagent (Invitrogen) and the concentration was measured with an ND-1000 spectrophotometer (Thermo Fisher Scientific, Wilmington, DE, USA). Totally, 2 µg of RNA was reversely transcribed into cDNA with the SuperScript™ III First–Strand Synthesis kit (cat. no. 11752–050, Thermo Fisher Scientific) following manufacturer's protocol. Real time quantitative PCR (qPCR) was performed for evaluation of Nox4 mRNA level. The PCR reaction system was 1× SYBR Green PCR Master Mix (Bio-Rad, Hercules, CA, USA), 1.5 μl of cDNA and 0.2 μM of Nox4 primers (forward: 5′- gactttacaggtatatccggagcaa–3′, reverse: 5′–tgcagatacactgggacaatgtaga–3′, 152 bp). The housekeeping gene glyceraldehyde 3-phosphate dehydrogenase (GAPDH) was used as the internal control (forward: 5′-accacagtccatgccatcac-3′, reverse: 5′-ggccatgccagtgagcttcc-3′, 171 bp). The amplification was carried out by an initial denaturation at 95°C for 5 min followed by 40 cycles of 95°C for 30 s, 60°C for 30 s and 72°C for 30 s. The mRNA level of Nox4 relative to GAPDH was calculated by using the 2^-ΔΔCt^. The fold change relative to the controls was presented and compared.

### Western blotting

Total cellular protein was extracted with the RIPA Lysis Buffer freshly supplemented with the Protease and Phosphatase Inhibitor cocktail (Sigma-Aldrich). Protein concentration was quantitated using the Bicinchoninic Acid (BCA) Protein Assay kit (Pierce Thermo Fisher Scientific, Rochester, MN, USA). A total of 50 µg protein was separated on 10% SDS–PAGE, and transferred to a nitrocellulose membrane (Abcam). Non-specific binding was blocked for 1 h in 5% BSA. Primary antibodies were incubated for overnight at 4°C, followed by 1 h incubation with HRP-conjugated goat anti-rabbit or mouse IgG (cat. no. 31340 and 31460, 1:10,000; Invitrogen). GAPDH was used as the loading and endogenous control. The primary antibodies were removed using Restore™ Western blotting Stripping Buffer (Thermo Fisher Scientific) for re-blotting the other primary antibody. The signals were detected with an ECL Substrate (Thermo Fisher Scientific), and quantified with ImageJ software (version 1.51 s; NIH, Bethesda, MD, USA). Total protein level was normalized to GAPDH, and the phosphorylated protein level was normalized to its total protein. Data are presented as the fold change relative to the controls.

### Intracellular cAMP detection

The intracellular cAMP concentration was measured with the Colorimetric cAMP ELISA Kit (Cell Biolabs, Inc., San Diego, CA, USA) according to the manufacturer's protocol. Briefly, cells were lysed by addition of 1 ml pre-cold Lysis Buffer in 10 cm plate. After incubation on ice for 20 min, cells were scraped off, transferred to a centrifuge tube and centrifuged at 12,000 rpm for 10 min. The supernatant was collected, and protein concentration was determined. Totally, 25 µg protein was used for assay according to the instructions, including two blank wells for background subtraction. The reaction was stopped by adding 100 µl of Stop Solution into each well. Results were read immediately at 450 nm on a microplate reader (BioTek, Beijing, China). cAMP concentration was calculated with the value of OD450 nm according to the standard curve.

### PKA activity detection

PKA activity was measured with the Protein Kinase A Colorimetric Activity Kit (cat. no. EIAPKA, Invitrogen) according to the manufacturer's instructions. Briefly, samples were applied to the microtiter plate immobilized with PKA substrate. In the presence of ATP, the immobilized PKA substrate was phosphorylated and then detected with rabbit anti-phospho-PKA substrate antibody in combination with peroxidase labelled rabbit IgG. After a short incubation and wash, peroxidase substrate was added and the intensity of the color developed was directly proportional to the amount of PKA in the samples and standards. The optical signal was read at 450 nm on a microplate reader (BioTek). Data are presented as the fold change relative to the controls.

### Intracellular ROS detection

The intracellular ROS level was measured with the peroxide-sensitive fluorescent probe 2′,7′-dichlorodihydrofluorescin diacetate (DCFDA) according to the instructions of the DCFDA Cellular ROS Detection Assay (Abcam). DCF fluorescence was detected at excitation and emission wavelengths of 488 and 520 nm on a microplate reader (BioTek). Data are presented as the fold change relative to the controls.

### Statistical analysis

Data are presented as the mean±standard deviation (s.d.). Statistical analysis was performed with One-way ANOVA with the Tukey's *post-hoc test* (GraphPad Prism6.0, GraphPad Software, Inc., La Jolla, CA, USA). *P*<0.05 was considered to have significant difference.
